# Chronic cholecystitis in the pediatric population: an underappreciated disease process 

**Published:** 2017

**Authors:** Brian P Blackwood, Julia Grabowski

**Affiliations:** 1 *Department of Pediatric Surgery, Ann and Robert H. Lurie Children’s Hospital of Chicago, Chicago, IL, USA*; 2 *Department of General Surgery, Rush University Medical Center, Chicago, IL, USA*

**Keywords:** Pediatric, Cholecystitis, Diagnosis, Intervention, Timing

## Abstract

**Aim:**

We hypothesize that chronic cholecystitis accounts for the majority of inflammatory diseases in the pediatric population and it is difficult to predict with preoperative ultrasound.

**Background:**

Despite the increase in gallbladder disease, there is a paucity of data on pediatric cholecystitis. Most pediatric studies focus on cholelithiasis and biliary dyskinesia rather than inflammatory gallbladder disease.

**Methods:**

We performed a single center retrospective review of all patients who underwent cholecystectomy from 1/1/10 – 1/1/15. Relevant data was extracted, including age, sex, acute vs. chronic presentation, duration of symptoms, preoperative imaging findings, and surgical pathology results.

**Results:**

Out of the 170 patients identified, there were 129 (75.9%) females and 41 (24.1%) males. The average age was 14 years (range 4-23 years). Seventy-six patients presented with acute symptoms with an average duration of pain of 2 days. Ninety-four patients presented with chronic symptoms and had an average duration of pain of 7.4 months. Eight patients (4.7%) had preoperative ultrasound that suggested inflammation, while the remaining showed only cholelithiasis. Pathology revealed chronic cholecystitis in 148 (87.1%). Among those who had pathologic evidence of chronic cholecystitis, preoperative inflammation was seen in only 5 patients (3.3%).

**Conclusion:**

Chronic cholecystitis accounts for the majority of pediatric inflammatory diseases. These data suggest that most pediatric patients experience episodes of inflammation prior to cholecystectomy. Underappreciated gallbladder inflammation may delay surgical referral, increase emergency department and primary doctor visits, and lead to more difficult operations. Surgeons should consider early cholecystectomy when cholelithiasis and symptoms are present.

## Introduction

 Cholecystitis is an inflammatory disease of the gallbladder, often related to cholelithiasis. Though an increase in laparoscopic cholecystectomies for pediatric gallstone disease has been noted over the past few decades, there is a paucity of data on pediatric cholecystitis (1-5). Studies in the literature related to pediatric gallbladder disease tend to focus on cholelithiasis secondary to hemolytic disorders as well as biliary dyskinesia, while there is little discussion about pediatric acute or chronic cholecystitis (1-4). 

Our institution has noted an increased number of gallbladder specimens from our pediatric patients with evidence of chronic inflammation. Despite thorough preoperative evaluations, including an ultrasound, most operations were performed for symptomatic cholelithiasis without mention of concern for cholecystitis. We, therefore, hypothesize that chronic cholecystitis accounts for the majority of inflammatory diseases in the pediatric population and it is difficult to predict with preoperative ultrasound.

## Methods

After obtaining IRB approval, a single-center retrospective review was performed at our free-standing university children’s hospital. The electronic medical record was queried for all pediatric patients who underwent a cholecystectomy during the time period from January 1st, 2010 to January 1st, 2015. The electronic medical records were queried for procedure codes consistent with cholecystectomy (47600, 47562, 47563, 47564, 47600, 47605, 47610, 30221071, 30221071, 30221996, 30221255, 30221265, and 70225013). 

Patients were included if they had undergone a cholecystectomy in the designated time period and had preoperative symptoms or clinical findings suggestive of an inflammatory biliary process. Preoperative symptoms that were considered to be consistent with a possible inflammatory process included abdominal pain, subjective fevers and chills, nausea, emesis, and inability to tolerate a diet. Clinical findings considered to be consistent with an inflammatory biliary process included right upper quadrant or epigastric tenderness, fever, an elevated white blood cell (WBC) count, or radiographic imaging (ultrasound, CT, or HIDA) that indicated an inflammatory process. Patients who underwent HIDA scan had normal appearing preoperative ultrasound but persistent symptoms. Radiologic indications of acute inflammation included pericholecystic fluid, gallbladder wall thickening, or evidence of an impacted stone. All preoperative imaging was reviewed by both the Pediatric Radiology Service and the Pediatric Surgery Service. Patients were included only if they had postoperative pathology results for final diagnosis. Pathologic specimens were diagnosed as chronic cholecystitis if they had findings of chronic inflammation such as gallbladder wall fibrosis and increased vascularity without signs of acute inflammatory changes. Patients were excluded if they underwent cholecystectomy for a non-inflammatory indication or as part of a more complicated procedure such as biliary atresia and liver transplant or to resect a choledochal cyst.

Records were reviewed and relevant data was extracted, including age, sex, acute vs. chronic presentation, duration of symptoms, WBC, preoperative imaging findings, surgical procedure and surgical pathology results. Operative time and major complications were recorded from the operative record.

Graphs and tables were generated using Excel. Statistical analysis was completed with Students T-Test or ANOVA where applicable using Graph Pad Prism 6 Software. Differences were considered significant at p<0.05.

## Results

In the 5-year period studied, 170 patients were identified who underwent a cholecystectomy and met the previously mentioned inclusion criteria. A total of 167 cholecystectomies were performed laparoscopically and 3 were performed with an open approach. The 3 open cases were performed due to patients having an extensive abdominal surgical history. There were no conversions from laparoscopic to open. The majority of patients (129 – 75.9%) were female and 41 (24.2%) were male. The ages of these patients ranged from 4 to 23 years, and the average age of all the patients was 14 years. 

All of the patients studied presented with complaints of abdominal pain, and they all had undergone a preoperative ultrasound of the gallbladder to assess for inflammatory or obstructive biliary disease. Seventy-six patients (44.7%) presented with acute onset of symptoms. These patients had an average duration of symptoms of 2 days. Ninety-four patients (55.3%) presented with chronic symptoms and these patients had an average duration of symptoms of 7.4 months.

**Figure 1:**
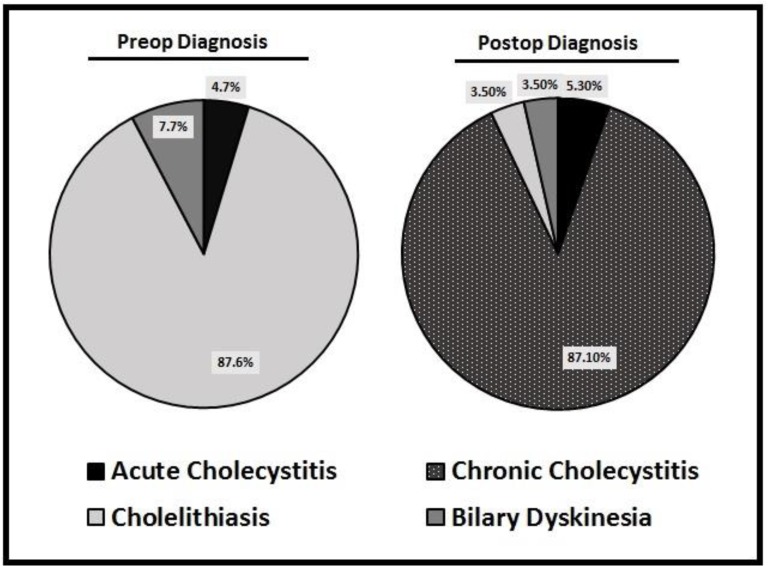
Preoperatively, 87.6% of patients had been diagnosed with cholelithiasis and very few (4.7%) had a diagnosis with concern for inflammatory disease. However, postoperatively, pathology revealed that actually 87.1% of patients had a diagnosis of chronic cholecystitis

Of these patients, only 8 (4.7%) had ultrasound findings consistent with inflammation. The vast majority (87.6%) showed cholelithiasis without any signs of inflammation preoperatively and 7.7% had findings consistent with biliary dyskinesia (positive preoperative HIDA scan and no pathologic signs of inflammation). However, when the pathology was reviewed, it was revealed that 148 patients (87.1%) actually had pathologic evidence of chronic cholecystitis (Figure 1). Looking only at these patients with pathologic evidence of chronic cholecystitis, only 5 (3.5%) had preoperative ultrasound showing signs of inflammation of the gallbladder. The remaining pathology revealed cholelithiasis without inflammation or normal gallbladder (12 patients), acute cholecystitis (9 patients), and a gallbladder polyp (1 patient) (Table 1). 

**Table 1:** Patient characteristics reveal the majority of patients to be female with and an average age of 14. The patients presented with both acute (44.7%) and chronic presentations (55.3%).

Gender:	
	41 (24.1%) 129 (75.9%)
Ave Age (yr):	14 (4-23)
Presentation:	
Acute Symptoms (N) Chronic Symptoms (N)	76 (44.7%) 94 (55.3%)
Operation:	
Laparoscopic Cholecysatectomy (N) Open Cholecystectomy (N)	167 (98.2%) 3 (1.8%)
Preop Diagnosis	
Acute Cholcystitis (N) Chronic Cholecystitis (N) Cholelithiasis (N) Biliary Dyskinesia (N)	8 (4.7%) 0 (0%) 149 (87.6%) 13 (7.7%)
Postop Path Diagnosis	
Acute Cholcystitis (N) Chronic Cholecystitis (N) Cholelithiasis/Biliary Dyskinesia (N) Gallbladder Polyp (N)	9 (5.3%) 148 (87.1%) 12 (7.1%0 1 (0.5%)

The average WBC for the patients with a pathologic diagnosis of chronic cholecystitis was 9.9 and was not significantly different from all other patients (p=0.50). There was no difference in operative time between those with inflammation and those without and no major complications were noted in either group (0.053). 

## Discussion

In the adult population, cholecystitis has been managed surgically for many decades (5). However, as previously stated, a rise has been noted in the number of cholecystectomies performed in the pediatric population in recent years (6, 7). Walker et al. reported that this rise in cholecystectomies can be attributed to an increase in biliary dyskinesia and other non-hemolytic diseases (8). This study also showed an increase, specifically in the incidence of cholecystitis in the pediatric population from 1997 – 2009, but it does not elaborate further on the nature of the cholecystitis (8). Another theory for the increase in pediatric gallstone disease is that it is a result of the increasing incidence of obesity, a known risk factor for cholecystitis in the adult population (9-13). Walker et al. also found that 53% of the patients studied were found to be either obese or overweight.

These recent findings prompted us to investigate the etiology behind the cholecystectomies performed at our own institution. Specifically, we wanted to analyze those patients who underwent cholecystectomy for inflammatory disease of the gallbladder to better understand the pathophysiology that is occurring in this population. Our 5-year review of laparoscopic cholecystectomies has shown chronic cholecystitis to be a much more prevalent disease than previously described. Interestingly, in our study, the large majority of patients (87.1%) were found to have pathology results consistent with chronic cholecystitis, despite the fact that 44.7% of patients presented with acute onset of symptoms and only 55.3% presented with a history of chronic symptoms. 

While there is a vast collection of literature on cholecystitis in the adult patient population, there is surprisingly very little literature on cholecystitis in the pediatric population. With an increasing incidence of pediatric cholecystitis, it is imperative that we understand completely the pathophysiology occurring in order to better treat these children. In the adult literature, there is evidence that early cholecystectomy in the setting of acute cholecystitis is superior to delayed cholecystectomy (14-19). These studies cite significant improvement in morbidity and length of hospital stay when early intervention is performed in the setting of acute cholecystitis. Furthermore, there is evidence of reduced hospital costs when acute cholecystitis is treated with cholecystectomy in the first 24 hours of presentation (20). 

It is important toWe can take the abundance of knowledge data we have from in the adult literature on the treatment of acute cholecystitis and extrapolate to the pediatric population. How the pediatric patient with symptomatic gallstones should be managed. We know from our studyOur study demonstrates that the majority of pediatric patients have chronic cholecystitis, regardless of whether they present with acute or chronic symptoms. The adult literature clearly shows that there are benefits from early intervention on acute cholecystitis, likely from an increased inflammatory response as time progresses. Knowing this, it is reasonable to consider early intervention on children presenting with symptoms of symptomatic cholelithiasis.

Data from the adult literature suggest that chronic cholecystitis is not simply a result of having gallstones since many patients with longstanding gallstones do not have pathologic evidence of chronic inflammation. Autopsy studies have shown that up to 33% of patients may have gallstones on post-mortem examination and 39% of those patients have normal gallbladders on histologic examination (21). Chronic cholecystitis more likely results from prolonged or recurrent episodes of acute inflammation and, though most often found in the context of cholelithiasis, it can also be present in patients without gallstones (22-24). A previous study examining biliary dyskinesia in children has better defined chronic cholecystitis based on a histopathologic grading system (25). This is a useful tool in analyzing the pathophysiology behind chronic cholecystitis in the pediatric population. Unfortunately given that our study is a retrospective review, we were not able to classify our patients’ pathologic findings in such detail. We also know from the adult literature that gallstones and chronic cholecystitis are independent risk factors for developing gallbladder malignancy (26). This knowledge provides an even stronger case for intervening earlier on symptomatic pediatric patients. 

Our study also identified another interesting finding in the inaccuracy in the use of preoperative ultrasound in the diagnostic workup of pediatric patients for inflammatory gallstone disease. In our study. 95.3% of patients underwent a preoperative ultrasound that was read as cholelithiasis without signs of either acute or chronic inflammation. However, when we analyzed our pathology results, 157 patients (92%) had pathologic evidence of inflammation (87.1% chronic, 5.2% acute). This represents a sensitivity of approximately 5%, which is not diagnostically helpful useful as the number of pediatric patients with cholecystitis increases. A recent study found that ultrasound findings have sensitivities as low as 6% for cholecystitis in the pediatric population compared to the adult population where sensitivities are as high as 96% (24, 27-30). This study also had similar findings to our study in that 83% of patients had a pathologic diagnosis of chronic cholecystitis while only 8% had acute cholecystitis.

The low sensitivity of ultrasound in diagnosing pediatric cholecystitis and the high incidence of patients with findings of chronic cholecystitis indicate a slightly different disease process and progression in the pediatric patient population compared to the adult population. As suggested by Tsai et al., these findings indicate that pediatric patients undergo multiple episodes of inflammation that are not significant enough to be seen radiographically, yet still produce enough pain that these patients present to the hospital for evaluation. As a result of this more indolent course, physicians may be delaying appropriate surgical intervention. This in turn may be increasing emergency department and primary care visits, adding to the overall healthcare costs.

We postulated that perhaps the chronic inflammation would lend itself to a longer operative time, a high number of conversions from laparoscopic to open or a high rate of complications. This did not prove to be true which may be due to the laparoscopic experience that pediatric surgeons currently obtain during adult surgery training.

A limitation of our study is that it is a retrospective study at a single institution. As such the criteria that other institutions use in their assessment of cholecystitis, both clinically and radiographically, may vary. Institutional preoperative imaging sensitivity may also vary slightly based on the expertise of those performing and interpreting the imaging studies. Additionally, as this is a retrospective review at a large metropolitan children’s hospital, there may be some selection bias and our cohort may not accurately represent the general pediatric population. Also, we were unable to capture retrospectively how many times the chronic patients presented (to the ED or the Clinicclinic) before undergoing cholecystectomy. This would be an interesting data point to collect in the future study of pediatric inflammatory gallbladder disease.

In conclusion, in our experience, chronic cholecystitis accounts for the majority of the inflammatory diseases seen in the pediatric patient population. These data suggest that most pediatric patients experience multiple episodes of inflammation prior to cholecystectomy. Underappreciated inflammation of the gallbladder may delay surgical referrals and increase emergency department and primary doctor visits. Though our data did not prove this, in certain patients, chronic inflammation could make operative intervention more difficult, increasing the risk of complications. Surgeons should consider early cholecystectomy when cholelithiasis and symptoms are present.

## Conflict of interest

The authors declare that they have no conflict of interest.
